# Common strategies in empirically supported psychological interventions for alcohol use disorders: A meta‐review

**DOI:** 10.1111/dar.13550

**Published:** 2022-09-22

**Authors:** Abhijit Nadkarni, Alessandro Massazza, Rahul Guda, Luanna T. Fernandes, Ankur Garg, Mehak Jolly, Lena S. Andersen, Urvita Bhatia, Sergiy Bogdanov, Bayard Roberts, Wietse A. Tol, Richard Velleman, Quincy Moore, Daniela Fuhr

**Affiliations:** ^1^ Department of Population Health London School of Hygiene and Tropical Medicine London UK; ^2^ Addictions Research Group, Sangath Goa India; ^3^ Department of Health Services Research and Policy London School of Hygiene and Tropical Medicine London UK; ^4^ Department of Public Health University of Copenhagen Copenhagen Denmark; ^5^ Centre for Mental Health and Psychosocial Support, National University of Kyiv‐Mohyla Academy Kyiv Ukraine; ^6^ HealthRight International New York New York USA; ^7^ Athena Research Institute, Vrije Universiteit Amsterdam Amsterdam the Netherlands; ^8^ Department of Psychology University of Bath Bath UK; ^9^ Leibniz Institute for Prevention Research and Epidemiology Bremen Germany; ^10^ Health Sciences University of Bremen Bremen Germany

**Keywords:** alcohol use disorders, meta‐review, psychological treatments

## Abstract

**Issues:**

Despite the large number of effective psychological interventions for alcohol use disorders (AUD), there is still a lack of clarity concerning the strategies that make these interventions effective.

**Approach:**

The overall goal of this review was to identify, examine and synthesise the information about common strategies from evidence‐based psychological interventions for AUDs by conducting a review of systematic reviews, that is, a meta‐review. We isolated the relevant primary studies from eligible systematic reviews and extracted information about the interventions from these studies to understand the strategies used. Analysis was restricted to narrative summaries.

**Key Findings:**

Thirteen reviews were eligible for inclusion in our meta‐review. Of these, eight demonstrated the effectiveness of a range of psychological interventions—behavioural couples therapy, cognitive behaviour therapy combined with motivational interviewing, brief interventions, contingency management, psychotherapy plus brief interventions, Alcoholics Anonymous and 12‐step treatment programs, family‐therapy or family‐involved treatment, and community reinforcement approach. The most commonly used component strategies in effective interventions for AUDs included assessment, personalised feedback, motivational interviewing, goal setting, setting and review of homework, problem solving skills and relapse prevention/management.

**Implications:**

Evidence about commonly used strategies in evidence‐based psychological interventions for AUDs offer the possibility of creating menu‐driven interventions that can be tailored to respond to individual client needs and preferences in different contexts.

## 1 INTRODUCTION

Alcohol use is a major contributor to mortality and disability, with 3 million alcohol‐attributable deaths and 131.6 million disability‐adjusted life years lost globally [1]. In the Global Burden of Disease Study 2017, alcohol use was also identified as a leading risk factor for premature death and disease burden among people aged 15–49 years old [2]. Despite substantial variation across contexts, the alcohol‐attributable burden of disease has been shown to be highest among countries with a low human‐development index, including eastern Europe, and western, central and eastern sub‐Saharan Africa regions [1]. Alcohol use disorders (AUD) are one of the most prevalent mental, neurological and substance use disorders globally, affecting 8.6% of men and 1.7% of women [3].

There is substantial evidence for psychological interventions that are effective in treating AUDs [4]. These interventions include motivational interviewing (MI), brief interventions, cognitive behavioural therapy (CBT), peer‐support interventions such as 12‐step treatment programs, contingency management and community reinforcement approaches [5, 6, 7, 8, 9, 10]. Despite the large number of effective interventions, there is still a lack of clarity concerning the active ingredients that make these interventions effective. Active ingredients have been defined as ‘those aspects of an intervention that drive clinical effect, are conceptually well defined, and link to specific hypothesised mechanisms of action’ [11]. The lack of a clear understanding concerning active ingredients in AUD interventions is reflected in the fact that evidence‐based AUD interventions often have similar degrees of effectiveness when directly compared to one another [12].

The similar degree of effectiveness between different evidence‐based psychological interventions for AUD may suggest that, although interventions are differently labelled, there are in fact some common strategies that underpin these interventions and that largely drive their effectiveness. This hypothesis is not unique to AUDs but has been suggested as cutting across psychological interventions for other conditions [13]. It posits that factors common across therapies (e.g., therapeutic alliance and empathy) are largely influential in driving effectiveness [14]. Hence, identifying strategies that are common to effective AUD interventions represents one way of understanding the components of an intervention that make a difference in managing AUDs. This would allow us to maximise clinical effectiveness of new interventions by incorporating appropriate strategies that are more likely to lead to change in drinking behaviours. Consequently, the overall goal of this review was to identify, examine and synthesise the information about common strategies of evidence‐based psychological interventions for AUD.

## 2 METHODS

### 2.1 Design

Review of systematic reviews, that is, meta‐review. The protocol for the review was registered a priori on PROSPERO (CRD42020209109).

### 2.2 Eligibility criteria

We did not have any limits on year of publication, setting, delivery platform (e.g., primary care, hospital) and country; and only included reviews published in English. We included systematic reviews with meta‐analyses of randomised controlled trials and/or non‐randomised trials. Reviews were included if they synthesised interventions for adults (≥18 years) of any gender with any type of AUD (e.g., harmful drinking, alcohol abuse, alcohol dependence) as defined by the source review or an AUD co‐morbid with any other mental/physical health condition or other substance use disorder. An intervention was eligible if it was a psychological intervention package delivered by humans, designed specifically to address AUD in individuals or groups, and was delivered by itself or in combination with a pharmacological intervention. Any transdiagnostic intervention package which addressed AUD as one of the target conditions was also eligible. Treatment as usual or usual care, enhanced usual care, other psychological treatment and pharmacological treatment were all eligible as control conditions. Reviews were eligible if one or more of the following outcomes were used to determine effectiveness: drinking patterns (e.g., quantity, frequency, intensity), remission, standardised tools measuring drinking (e.g., Alcohol Use Disorders Identification Test), impact of drinking measured using standardised tools (e.g., Short Inventory of Problems) and global functioning, disability or quality of life measured using standardised tools (e.g., World Health Organization Disability Assessment Schedule).

We excluded non‐systematic reviews such as literature reviews, scoping reviews, reviews in which quantitative outcomes reporting effectiveness were not presented, systematic reviews without an accompanying meta‐analysis, and realist reviews. We excluded the following interventions: any psychological intervention not specifically targeting AUD even if drinking outcomes were measured, policy interventions, educational or training interventions, interventions targeting only family members of those with AUD, universal population level interventions, primary and secondary prevention interventions, pharmacological interventions only, and mHealth interventions unless technology was used as a delivery platform for delivery of interventions by humans (e.g., telephone counselling).

### 2.3 Data sources and searches

We searched the following databases: Cochrane Library, MEDLINE, Embase, PsycINFO, Global Health, Cumulative Index to Nursing and Allied Health Literature, Latin American and Caribbean Health Sciences Literature and African Journals Online. We limited our results to publications indexed as review articles in the database. If that functionality was not available in the database, then we used the relevant search terms for ‘review’ (e.g., systematic review, meta‐analysis) in the search strategy. Our key search concepts included ‘alcohol use disorders’ and ‘psychological intervention’ (e.g., psychosocial intervention, cognitive behaviour therapy, family therapy). The detailed search strategy is outlined in Data S1 (Supporting Information). We also inspected the reference lists of selected reviews to identify any additional potential reviews.

### 2.4 Data extraction and analysis

The search returns were uploaded into Covidence (www.covidence.org) and de‐duplicated. Two reviewers (AG and AM) independently inspected the titles and abstracts of the reviews. If the title and abstract did not offer enough information to decide about eligibility, the full paper was retrieved to ascertain eligibility for inclusion. The full papers were independently inspected for eligibility by two reviewers (LTF and AM). If there was any disagreement between the two reviewers regarding inclusion, a third reviewer (AN) resolved the conflict. Following Preferred Reporting Items for Systematic Reviews and Meta‐Analyses (PRISMA) guidelines, a record was made of the number of papers retrieved, number of papers excluded and the reasons for their exclusion. A formal data extraction form was designed to extract data relevant to the study aims. The data extraction tool was designed to collect data within the following broad domains: number of trials included in meta‐analysis, eligibility criteria for inclusion, study designs, intervention, control conditions, study population, type of AUD, total sample size, outcomes, intervention effect and moderators/mediators. Two reviewers (LSA and AM) independently conducted a quality assessment of the included reviews using the AMSTAR 2 criteria for systematic reviews of randomised and non‐randomised studies of health‐care interventions [15].

We extracted individual trials from these reviews. After excluding duplicate trials, we screened the rest for determining eligibility for extraction of intervention strategies. When screening individual trials, we followed the same inclusion and exclusion criteria which we applied to the meta‐analyses as described above. Therefore, we only included trials which tested psychological interventions for AUD delivered by humans (e.g., if a meta‐analysis included trials for AUD but also trials aimed at other substances we only extracted data from trials focusing on AUD).

From the eligible primary studies we extracted information about the interventions to understand the intervention strategies. Data on common strategies was identified by extracting descriptions of the intervention's content. This data was extracted from the primary paper or the relevant accompanying secondary paper which described the intervention. Analysis was restricted to narrative synthesis.

## 3 RESULTS

### 3.1 Eligible reviews

**FIGURE 1:**
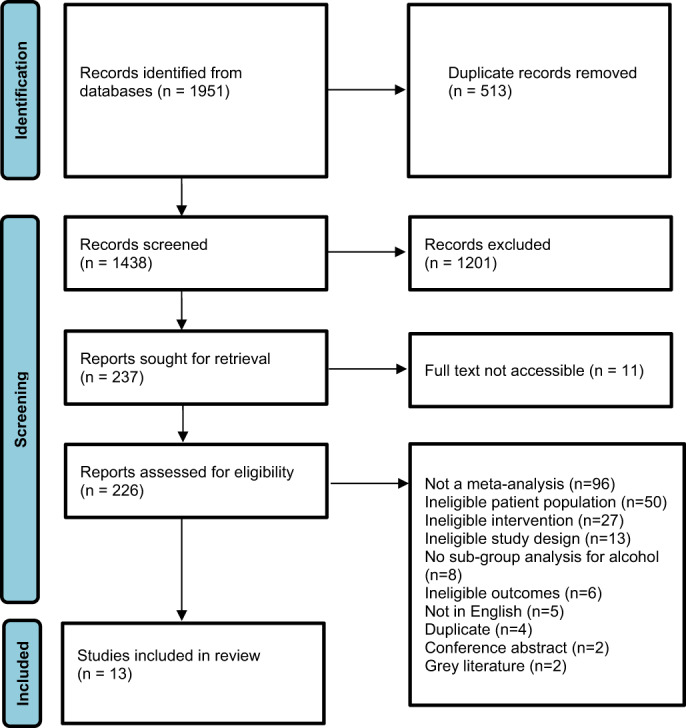
PRISMA flow chart describing the screening process

The search of the databases returned 1951 references. After excluding 513 duplicates, we screened titles and abstracts of 1438 studies. We excluded 1201 studies as they were irrelevant to our review, we were not able to retrieve 11 full texts, and assessed the full text of 226 studies for eligibility. We excluded 213 studies for various reasons, the most common being that there was no meta‐analysis (*n* = 97) and the sample did not have any type of AUD (*n* = 51). Thirteen reviews were eligible for inclusion in this meta‐review [5, 6, 7, 8, 9, 10, 16, 17, 18, 19, 20, 21, 22]. Figure 1 summarises the screening process described above.

Table 1 summarises the systematic reviews included in this meta‐review. The number of studies meta‐analysed in the reviews we identified ranged from 4 [22] to 137 [9] The included reviews differed from each other in several ways as summarised below.

**TABLE 1:** Systematic reviews and meta‐analyses included in the meta‐review

Author, year	Number of trials included in meta‐analysis	Intervention	Control	Measure of AUD	Total sample size of meta‐analysis	Quality assessment
Agosti, 1994	15	Various	Not specified	Participants in 7 out of the 15 studies met criteria for DSM‐III criteria for alcohol dependence. The other studies did not use DSM‐II criteria, but all patients had clinically significant alcohol‐related problems.	1,912	Critically low quality
Agosti, 2012	6 (4 relevant)	CBT	Non‐CBT psychosocial treatment	Current alcohol dependence as per DSM‐III‐R or DSM‐IV	1,008 (4 relevant trials)	Low quality
Edwards, 1995	21	Family therapy	Various	Alcoholism	Not reported	Critically low quality
Elzerbi, 2015	28	Brief intervention	Various	Non‐treatment seeking hazardous or harmful drinking. Harmful drinking was regular average consumption of >40 g of alcohol per day for women and >60 g per day for men.	13,025	Moderate quality
Gao, 2018	137	Psychotherapy, contingency management, brief intervention	No treatment, other types of treatment	AUD	27,282	Moderate quality
Hettema, 2005	72 (31 relevant)	MI ± feedback, MI + other active treatment	Control condition without elements of MI	Alcoholism, alcohol abuse, addictive behaviours	14,267 (for all outcomes)	Critically low quality
Kelly, 2020	27	Alcoholics Anonymous, 12‐step facilitation	Other interventions, no treatment	AUD, alcohol abuse or alcohol dependence, as defined using standardised criteria (i.e., DSM 4th and 5th edition, ICD‐9 and 10, and validated screening and diagnostic tools)	10,565	High quality
Kownacki, 1999	21	Alcoholics Anonymous	Various	Axis I diagnosis of alcohol misuse or alcohol dependence, or who were clearly identified primarily as alcoholics, alcohol misusers or problems drinkers	Not reported	Low quality
Powers, 2008	12 (8 relevant)	Behavioural couples therapy	Active or inactive controls	Various	499 couples for relevant studies (754 in total)	Critically low quality
Ray, 2020	30 (15 relevant)	CBT	Usual care, another specific therapy plus pharmacotherapy, usual care and pharmacotherapy alone	Meeting criteria for AUD (DSM‐III to 5th edition) or problematic use	Not reported	Low quality
Riper, 2014	12	CBT + MI	Treatment as usual	AUD, abuse or dependence	1,721	Low quality
Roozen, 2004	11 (2 relevant)	CRA	Usual care	Alcohol dependence (DSM‐IV)	343 (for relevant studies)	Moderate quality
Roozen, 2006	24 (17 relevant)	CBT, group psychotherapy	Various	Alcohol dependence (DSM‐III‐R or DSM‐IV)	Not reported	Moderate quality

Abbreviations: AUD, alcohol use disorders; CBT, cognitive behaviour therapy; CRA, Community Reinforcement Approach; DSM, Diagnostic and Statistical Manual of Mental Disorder; ICD, International Classification of Diseases; MI, motivational interviewing.

Powers et al. reviewed 12 studies of which only eight focussed on AUDs [19]. In Riper et al. the meta‐analyses made 15 comparisons, of which 10 focused on comorbid alcohol use and depression, and 1 only on AUD [7]. In Hettema et al. of the 72 studies included in the review, 31 were about AUD [6] and in Ray et al. of the 30 studies included in the review, 15 focussed on AUD [20].

### 3.2 Conceptualisation of AUDs in the eligible reviews

In the included papers, AUDs were variously described as alcoholism, alcohol dependence, harmful drinking and alcohol abuse/misuse. A range of outcomes were included in the meta‐analyses—quantity of use (e.g., grams of alcohol consumed per week) [5, 6, 7, 10, 20], frequency of use (e.g., % days abstinent) [6, 19, 20], intensity of use (e.g., % days heavy drinking) [8], intoxication (blood alcohol concentration) [6], abstinence [7, 8, 9, 17], relapse, consequences of use (e.g., Addiction Severity Index) [6, 8, 19] and relationship satisfaction [19].

### 3.3 Number and design of studies synthesised in the eligible reviews

Agosti et al. synthesised four trials that used naltrexone or placebo in combination with random assignment to CBT or non‐CBT psychotherapies [22]. Elzerbi et al. separately meta‐analysed interventions delivered in primary care (*n* = 20) and emergency departments (*n* = 8) [5]. The largest review had 137 studies but these also included pharmacotherapy‐only studies [9]. Kelly et al. reviewed 27 studies with varied study designs—21 randomised controlled trials/quasi‐randomised controlled trials, 5 non‐randomised studies of interventions and 1 purely economic study [8]. In Roozen et al., of the 11 studies included in the review, 2 of which measured number of drinking days as an outcome were added to the meta‐analysis [10]. Finally in Roozen et al., of the eight studies of psychosocial treatment, four each involved individual treatment and group psychotherapy [21].

### 3.4 Interventions

Some reviews focused on discrete therapeutic interventions such as behavioural couples therapy [19], CBT [22], Alcoholics Anonymous or 12‐step facilitation [8, 18], family therapy [17], MI [6], contingency management [9] and community reinforcement approach [10]. Some reviews examined broad psychosocial programs, brief interventions [5] and ‘psychotherapy’ which provided an eclectic mix of intervention strategies [9, 16]. Finally, some reviews examined a combination of psychotherapies such as CBT and MI [7], and MI added to other types of active treatment (e.g., cognitive therapy) [6]; and a combination of psychotherapy with pharmacotherapy [18].

### 3.5 Quality of included reviews

We assessed all 13 reviews for quality (Data S2). One review was deemed to be of high quality [8], five reviews of moderate quality [5, 9, 10, 20, 21], three reviews of low quality [7, 18, 22] and four of critically low quality [6, 16, 17, 19]. The ratings of the two independent reviewers matched for all reviews except for one. The discrepancies were discussed and agreement reached for all reviews. Common methodological shortcomings of the included reviews were: not providing an explicit statement highlighting that the methods were established prior to the conduct of the review, issues concerning the search (e.g., small number of databases searched, search strategy not reported), study selection and data extraction not conducted in duplicate, not providing a list of excluded studies, not considering potential impact of risk of bias in individual studies on the results, and not investigating possible publication bias.

### 3.6 Effectiveness

Five meta‐analyses failed to show effectiveness of the intervention under investigation [16, 18, 20, 21, 22] and no information on the individual trials was therefore extracted from them. The remaining eight meta‐analyses demonstrated the effectiveness of a range of interventions (Data S3) and were retained for data extraction from individual trials.

Powers et al. found behavioural couples therapy to be effective in addressing AUD when compared to active or inactive controls (Hedges *g* = 0.55 for the eight studies focusing on alcohol) [19]. Riper et al. found that CBT combined with MI was effective for adults with comorbid major depressive disorder and AUD in comparison with control (i.e., treatment as usual or active psychological treatment) (*g* = 0.17 for alcohol consumption at post‐treatment, *g* = 0.16 for alcohol consumption when only considering face‐to‐face interventions) [7]. Elzerbi et al. identified brief interventions as effective in reducing alcohol consumption among people with hazardous or harmful alcohol use when compared with control groups in both primary health care and emergency department settings at both 6 (mean difference; MD = 21.98 g/week in primary health care and MD = 17.97 g/week in emergency department) and 12 months follow‐up (MD = 30.86 g/week in primary health care and MD = 18.21 g/week in emergency department) [5]. Gao et al. identified in their direct meta‐analysis significant differences in abstinence rates for pharmacotherapy plus psychotherapy (odds ratio [OR] = 1.17), contingency management (OR = 1.30), brief interventions (OR = 1.06) and psychotherapy plus brief intervention (OR = 1.50) when compared to controls in treatment sessions [9]. Kelly et al. found Alcoholic Anonymous and 12‐step treatment programs (manualised) compared to treatments with different theoretical orientations (e.g., CBT), was effective in improving rates of continuous abstinence at 12 months (risk ratio = 1.21) and with similar effectiveness for percentage days abstinent at 12 months (MD = 3.03) (but more effective at 24 months, MD = 12.91 and 36 months, MD = 6.64), longest period of abstinence at 6 months (MD = 0.60), drinking intensity at 12 months (MD = −0.17) and alcohol‐related consequences at 12 months (MD = −2.88) [8]. Similarly, for the non‐manualised versions, Alcoholics Anonymous and 12‐step treatment programs performed as well as other clinical interventions in terms of proportion of participants that were completely abstinent at 3–9 months follow‐up (risk ratio = 1.71) and for drinking intensity at 9 months (MD = −1.76) and slightly better than other clinical interventions for percentage days of abstinence (MD = 3.00). Edwards and Steinglass found that family‐therapy or family‐involved treatment was marginally more effective than individual alcoholism treatment in increasing abstinence both in primary treatment/rehabilitation as well as in aftercare (i.e., maintenance or relapse prevention) [17]. Hettema et al. found that MI was effective in addressing alcohol abuse when compared with control conditions without elements of MI, yielding *d*_c_ values between −0.08 and 3.07, with a mean of 0.41 post‐treatment and 0.26 across all follow‐up points [6]. Roozen et al. found strong evidence for the community reinforcement approach being more effective than usual care in relation to the number of drinking days (weighted mean difference −0.94 in favour of community reinforcement approach) and conflicting evidence in relation to continuous abstinence in alcohol treatment [10].

The control groups in the trials included in the reviews were: (i) active psychological treatments such as CBT, interpersonal behaviour therapy, psychoeducation attention control treatment, alcohol‐focused spouse intervention, 12‐step facilitation and its variants, motivational enhancement therapy, interpersonal therapy, supportive psychotherapy, family therapy, contingency management, systematic desensitisation and insight therapy; (ii) non‐specified ‘psychotherapy’, both individual and group; (iii) usual care, both standard (e.g., psychosocial counselling, medication treatment) or non‐standard (e.g. ‘traditional program’); (iv) minimal intervention such as screening/assessment only, information leaflet, minimal advice and education; (v) pharmacotherapy—unspecified or specific (e.g., naltrexone); (vi) clinical management, primary care management, medical management; (vii) waiting list control; and (viii) no treatment. In a few cases, the control arm was not specified.

### 3.7 Trials from the included reviews

We extracted 254 individual trials from the eight reviews that showed positive effects for the interventions under investigation. After excluding 15 duplicate trials, we screened the rest for determining eligibility for extraction of intervention strategies. One hundred and seventy‐two trials were excluded for one or more of the following reasons—they tested interventions only for substances other than alcohol, only tested pharmacotherapy, sample had participants who drank heavily but did not have AUD (e.g., risky drinkers), not peer‐reviewed (e.g., dissertation), intervention was for a co‐morbid mental health condition and participants were not adults. The full text of one trial could not be retrieved despite having contacted the corresponding author and was therefore excluded. Sixty‐six trials tested psychosocial interventions for AUD and were used to extract data about intervention content.

### 3.8 Common strategies

Table 2 summarises the strategies used in the interventions based on how frequently they were reported in the 66 trials. The most commonly used strategies included those implemented in the initial stages of treatment (e.g., assessment, personalised feedback, goal setting), those implemented while ending treatment (e.g., relapse prevention/management) and those implemented through the course of the treatment (motivational interviewing, setting and review of homework and problem‐solving skills). The more commonly used strategies were behavioural (e.g., assertiveness training, alternative activities) compared to cognitive strategies (e.g., identifying and disputing distorted thoughts, cognitive self‐management techniques for reducing negative thoughts).

**TABLE 2:** Strategies used in evidence‐based interventions for managing alcohol use disorders

Reported in ≥10 trials	Reported in 5–9 trials	Reported in 2–4 trials	Reported in only 1 trial
Assessment (*n* = 17)	Communication skills (*n* = 9)	Relationship enhancement exercises (*n* = 4)	Decision making skills
Personalised feedback (*n* = 21)	Self‐monitoring (*n* = 8)	Psychoeducation (*n* = 2)	Dealing with accusation of relapse
Motivational interviewing (*n* = 18)	Changing social networks (*n* = 5)	Direct advice on reducing drinking (*n* = 2)	Enhancing self‐esteem
Goal setting (*n* = 11)	Emotional management (*n* = 5)	Daily mood monitoring (*n* = 2)	Stress management
Setting and review of homework (*n* = 10)	Handling drinking urges (*n* = 6)	Cognitive restructuring (*n* = 2)	Behavioural self‐control training
Problem solving skills (*n* = 12)	Pros and cons of drinking (*n* = 6)	Social skills training (*n* = 3)	Importance/confidence
Relapse prevention/management (*n* = 12)	Action planning/change plan (*n* = 6)	Relaxation training (*n* = 3)	Identifying and disputing distorted thoughts (cognitive restructuring)
	Assertiveness training (*n* = 7)	Abstinence contracting (*n* = 3)	Cognitive self‐management techniques for reducing negative thoughts
	Alternative activities (*n* = 8)	Anger management (*n* = 4)	
	Identifying high risk situations (*n* = 8)		
	Coping skills to deal with high‐risk situations (*n* = 8)		
	Drink refusal skills/handling peer pressure (*n* = 7)		
	Contingency management (*n* = 9)		
	Information booklet (*n* = 9)		

## 4 DISCUSSION

The most important finding of our review is that we have identified the strategies that most commonly feature in effective psychological interventions for AUDs—assessment, personalised feedback, motivational interviewing, goal setting, setting and review of homework, problem solving skill, and relapse prevention/management. The advantage of having such a range of strategies that work is that therapists can choose from a wide selection to pick the ones best suited to individual patients' needs, contexts and preferences. However, our findings do not allow us to draw conclusions on how these strategies can be brought together in a theoretically supported manner.

In 1936, Saul Rosenzweig (1907–2004) applied the concept of the Dodo Bird Verdict (‘Everybody has won, and all must have prizes’) to psychological treatments observing that all therapies result in comparable outcomes, and concluded that they probably worked through factors that were common to them all [23]. Subsequent research over the years has largely supported this observation that, for some mental health problems, different psychological treatments have comparable effects and do not significantly differ from one another [24, 25]. Psychological treatments for AUDs are not much different, with seminal studies which pitted different types of treatments demonstrating there were comparable outcomes for therapies such as motivational enhancement therapy, CBT, 12‐step facilitation and social behaviour and network therapy [26, 27].

In the absence of clear superiority of one psychological treatment over another, ‘transdiagnostic clinical interventions’ (interventions that apply the same treatment principles across mental health conditions without tailoring those to a specific diagnosis) [28] could be the potential way forward. There is substantial evidence demonstrating the equivalence or superiority of transdiagnostic approaches over diagnosis‐specific interventions for anxiety and depressive disorders [29, 30]. Such trans‐diagnostic approaches become particularly relevant for AUDs, as AUDs lie along a spectrum of severity and are characterised by heterogeneity of presentation and comorbidity with other mental health conditions. An appropriate transdiagnostic response to such a condition could be a hybrid intervention that combines universal strategies (all clients receive the same set of strategies that have the broadest applicability across diagnoses) with the modular approach, which provides a menu of relatively self‐contained functional strategies that operate independently, problem‐specific, and are delivered flexibly and tailored to the needs of each client. The key advantage of universal strategies is potential scalability as clinicians, especially non‐specialists, can be more easily trained compared to training in separate intervention protocols for different disorders. The modular approach on the other hand allows for a better goodness‐of‐fit between the strategy and the individual clinical presentation. However, a modular approach comes with multiple decision points and this might be challenging for non‐specialist health workers.

Such a hypothetical intervention arising from our findings could be made up of universal strategies drawn from those that most commonly feature in effective interventions (Table 2). The reasons why these could potentially apply to all clients regardless of severity of AUD and idiosyncrasies of individual presentation are clear. The ‘assessment’ will allow for understanding the extent of the problem and the subsequent ‘personalised feedback’ could initiate change in behaviour by correcting misperceptions and highlighting discrepancies about drinking, ‘motivational interviewing’ would promote behaviour change by helping clients to explore and resolve ambivalence. Furthermore, ‘goal setting’ will allow clarification of the drinking goals that the client wants to achieve and ways to achieve those, ‘setting and review of homework’ will ensure that skills learnt during therapy sessions are further strengthened between sessions, ‘problem solving skills’ will provide problem‐agnostic skills that can be applied across problems that either result in or result from the drinking. Finally, ‘relapse prevention/management’ provides tools that the client can use to sustain their changed drinking behaviour. The strategies that are next in the list (i.e., reported in 5–9 trials) can then be delivered through a modular approach to those clients who need them, for example, assertiveness training for those who succumb to peer pressure resulting in drinking or handling drinking urges for those whose drinking occurs primarily in response to internal or external cues. Finally, although we present some strategies separately because they are reported as such in the source trials there is an overlap in their functions in many clinical interventions, for example, ‘Motivational interviewing’ and ‘pros and cons of drinking’; ‘relapse prevention’ and ‘coping skills to deal with high risk situations’.

Our findings have several research and related clinical implications. Identifying strategies that are common across effective psychological treatments for AUD would assist research that aims to isolate the effectiveness of such specific strategies for particular outcomes. This would allow researchers to further address the question of what works for whom and under what conditions, and this can then be used to further elaborate the mechanisms to achieve optimal treatment outcomes.

The key strength of our review is the rigorous methodology that we have followed and the large number of trials from which we were able to extract data about the interventions; but it does have some limitations. The strategies that we extracted are limited by the descriptions of interventions in published articles and this might not reflect the entirety of the interventions. Also, because it was a review of reviews, we did not examine individual studies in‐depth and hence did not capture more information on the rationale, theoretical approach, design of the selected interventions and other issues related to their implementation (e.g., fidelity). Additionally, the heterogeneity of the samples in the studies within and across reviews limits the conclusions that we can draw. Very few of the final selected eight reviews were of high quality. Although that is not a methodological limitation of our meta‐review, it may potentially limit our findings (e.g., if the source trials did not consider potential impact of risk of bias in individual studies on their results). Our review includes studies extracted from reviews and the most recent review was from 2020 (consequently the extracted studies were older than that). Although it is unlikely that many new strategies would have been introduced in the interim, it is possible that studies could have been conducted in other/more settings with newer understanding of how these strategies operate in other contexts. Finally, we judged intervention success narrowly in terms of statistical significance which we know is an arbitrary criterion and intervention success is a function of both subjective and objective outcomes covering aspects beyond the clinical, for example, social functioning. Relatedly, it is quite possible that a strategy that we have included because it was a component of an effective intervention in one trial could also be a component of an intervention which was found to be ineffective in a different evaluation. Additionally, as we did not extract data from any trials in reviews with null findings, it is possible that we might have missed out on some individual trials with positive results in those reviews. Finally, a key limitation that is inherent to meta‐reviews is that synthesis at the level of reviews does not allow for adequate exploration of nuance such as a control condition in one trial being the primary intervention evaluated in a different trial. Further research is needed to understand how the interaction between different strategies, and also between strategies and context, determine why they work in some interventions and not in others.

## 5 CONCLUSION

Many strategies from psychological interventions for AUDs are associated with positive treatment outcomes, offering the possibility of creating menu‐driven interventions that can be tailored to respond to individual client needs and preferences. However, further studies are necessary to operationalise the efficient integration of these strategies into interventions based on a credible mechanism of change; and establish the effectiveness of such interventions.

## AUTHOR CONTRIBUTIONS

Each author certifies that their contribution to this work meets the standards of the International Committee of Medical Journal Editors.

## FUNDING INFORMATION

This work was supported by the National Institute for Health Research (NIHR) (using the UK's Official Development Assistance Funding) and Wellcome (grant reference number 219468/Z/19/Z) under the NIHR‐Wellcome Partnership for Global Health Research. The views expressed are those of the authors and not necessarily those of Wellcome, the NIHR or the Department of Health and Social Care.

## Supporting information


**Data S1** Search strategy (Designed for Medline. Search strategies for other databases were modelled on this one and adapted to meet the requirements of those databases)
**Data S2**. Quality assessment of included reviews
**Data S3**. Results of meta‐analyses from reviews included in this meta‐review
