# Supplementary material for: Common strategies in empirically supported psychological interventions for alcohol use disorders: A meta‐review
Source: Drug Alcohol Rev. 2022 Sep 22;42(1):94–104. doi: 10.1111/dar.13550 (PMC10087716; doi:10.1111/dar.13550)
Supplement: Supplementary file 1 — Data S1 Search strategy (Designed for Medline. Search strategies for other databases were modelled on this one and adapted to meet the requirements of those databases) Data S2. Quality assessment of included reviews Data S3. Results of meta‐analyses from reviews included in this meta‐review [file DAR-42-94-s001.docx]

**Appendix S1. Search strategy** (Designed for Medline. Search strategies for other databases were modelled on this one and adapted to meet the requirements of those databases).

1. Alcohol use disorder.tw
2. Dependent drink$.tw
3. Alcohol dependen$.tw
4. Problem drink$.tw
5. Heavy drink$.tw
6. Harmful Drink$.tw
7. Binge drink$.tw
8. Heavy Episodic drink$.tw
9. Unhealthy drink$.tw
10. Alcohol abus$.tw
11. Alcohol misus$.tw
12. **OR (1-11)**
13. Alcoholism/
14. Alcohol Drinking/
15. Alcohol Abstinence/
16. Alcohol-Related Disorders/
17. Alcoholic Intoxication/
18. Alcohol-induced disorders/
19. **OR (13-18)**
20. **12 OR 19**
21. Psychotherap*.tw
22. Therap*.tw
23. Counsel?ing.tw
24. Psychosocial intervention*.tw
25. Psychosocial treatment*.tw
26. Psychosocial therap*.tw
27. Psychosocial support.tw
28. Psychological intervention*.tw
29. Psychological treatment*.tw
30. Psychological therap*.tw
31. Psychological support.tw
32. Cognitive behavio?r therapy.tw
33. Behavio?ral activation.tw
34. Interpersonal therapy.tw
35. Motivational interviewing.tw
36. Motivational enhancement therapy.tw
37. 12-step facilitation.tw
38. Group therapy.tw
39. Cognitive behavio?ral coping skills therapy.tw
40. Brief intervention*.tw
41. Brief therapy.tw
42. Dialectical Behavio?r Therapy.tw
43. Rehabilitation.tw
44. Contingency management.tw
45. Family therapy.tw
46. Peer support.tw
47. Art therapy.tw
48. Mindfulness.tw
49. Relapse prevention.tw
50. Community reinforcement.tw
51. **OR (21-50)**
52. Psychotherapy/
53. Counselling/
54. **OR (52-53)**
55. **51 OR 54**
56. **20 AND 55**

**For databases that do not have the ‘review’ filter we have to run the search strategy till #56 above and then append the following to it:**

1. Systematic review.tw
2. Meta-analys#s
3. **OR (57-58)**
4. **56 AND 59**

**Appendix S2. Quality assessment of included reviews**

| Author, year | 1. Did the research questions and inclusion criteria for the review include the components of PICO? | 2. Did the report of the review contain an explicit statement that the review methods were established prior to the conduct of the review and did the report justify any significant deviations from the protocol? | 3. Did the review authors explain their selection of the study designs for inclusion in the review? | 4. Did the review authors use a comprehensive literature search strategy? | 5. Did the review authors perform study selection in duplicate? | 6. Did the review authors perform data extraction in duplicate? | 7. Did the review authors provide a list of excluded studies and justify the exclusions? | 8. Did the review authors describe the included studies in adequate detail? | 9. Did the review authors use a satisfactory technique for assessing the RoB in individual studies that were included in the review? | 10. Did the review authors report on the sources of funding for the studies included in the review? | 11. If meta-analysis was performed did the review authors use appropriate methods for statistical combination of results? | 12. If meta-analysis was performed, did the review authors assess the potential impact of RoB in individual studies on the results of the meta-analysis or other evidence synthesis? | 13. Did the review authors account for RoB in individual studies when interpreting/ discussing the results of the review? | 14. Did the review authors provide a satisfactory explanation for, and discussion of, any heterogeneity observed in the results of the review? | 15. If they performed quantitative synthesis did the review authors carry out an adequate investigation of publication bias (small study bias) and discuss its likely impact on the results of the review? | 16. Did the review authors report any potential sources of conflict of interest, including any funding they received for conducting the review? | Result (from online calculation) |
| --- | --- | --- | --- | --- | --- | --- | --- | --- | --- | --- | --- | --- | --- | --- | --- | --- | --- |
| Agosti, 1994 | No | No | No | No | No | No | No | No | No | No | No | No | No | No | No | No | Critically low |
| Agosti et al., 2012 | Yes | No | No | Yes | No | No | No | Partial yes | Yes | Yes | Yes | Yes | No | Yes | Yes | Yes | Low |
| Edwards & Steinglass, 1995 | Yes | No | Yes | No | No | No | No | Yes | Partial yes | No | No | No | Yes | No | No | No | Critically low |
| Elzerbi et al., 2015 | Yes | No | No | Partial yes | No | No | Yes | Yes | Yes | No | Yes | No | Yes | Yes | No | Yes | Moderate |
| Gao et al., 2018 | Yes | No | No | Partial yes | No | Yes | No | No | Yes | No | Yes | No | Yes | Yes | Yes | Yes | Moderate |
| Hettema et al., 2005 | Yes | No | No | No | No | Yes | No | Partial yes | Partial yes | No | No | Yes | Yes | Yes | No | No | Critically low |
| Kelly et al., 2020 | Yes | Yes | Yes | Partial yes | Yes | Yes | Yes | Yes | Yes | Yes | Yes | No | Yes | Yes | N/A | Yes | High |
| Kownacki & Shadish, 1999 | Yes | No | Yes | No | No | No | No | Partial yes | Partial yes | No | Yes | No | Yes | Yes | No | Yes | Low |
| Powers et al., 2008 | Yes | No | No | No | No | No | Partial yes | Partial yes | No | No | Yes | No | No | Yes | Yes | No | Critically low |
| Ray et al., 2020 | Yes | Partial yes | Yes | Partial yes | Yes | Yes | No | Partial yes | Yes | No | Yes | No | Yes | Yes | Yes | Yes | Moderate |
| Riper et al., 2014 | Yes | No | Yes | Partial yes | Yes | No | No | Partial yes | Partial yes | No | Yes | No | No | Yes | Yes | Yes | Low |
| Roozen et al., 2006 | Yes | No | No | Partial yes | No | Yes | Partial yes | No | Yes | No | Yes | No | Yes | Yes | No | No | Moderate |
| Roozen et al., 2004 | Yes | No | No | Partial yes | Yes | Yes | Partial yes | Partial yes | Yes | No | Yes | No | Yes | Yes | No | Yes | Moderate |

PICO, patient/population, intervention, comparison and outcomes.

**Appendix S3. Results of meta-analyses from reviews included in this meta-review**

| **Author, year** | **Intervention effect** |
| --- | --- |
| Agosti, 1994 | No |
| Agosti, 2012 | No |
| Edwards, 1995 | Difference between family-involved treatment and controls was 0.75, which was statistically significant. For family-involved behavioural treatment and rehabilitation the mean effect size was a significant 0.86. The average effect size for the outcome measure of abstinence between the family and non-family involved treatments was a significant 0.94 |
| Elzerbi, 2015 | *Primary care*  Statistically significant benefits of BI at 6 months (MD 21.98 g/week; 95% CI 37.40, 6.57; p = 0.005) and 12 months (MD 30.86 g/week; 95% CI 46.49, 15.23; p = 0.0001).  *Emergency department*  Statistically significant benefits of BI at 6 months (MD 17.97 g/week; 95% CI 29.69, 6.24; p= 0.003) and 12 months (MD 18.21 g/week; 95% CI 26.71, 9.70; p <0.0001). |
| Gao, 2018 | *Network meta-analysis*  BI vs control: OR 0.81  CM vs. control: OR 0.30  CM + psychotherapy vs control: OR 0.20  Pharmacotherapy + BI vs control: OR 2.30  Pharmacotherapy + psychotherapy vs control: OR 1.76  Psychotherapy vs control: OR 1.51  Psychotherapy + BI vs control: OR 1.82  *Direct meta-analysis*  Pharmacotherapy plus psychotherapy vs control: OR 1.178 (95% CI 1.002–1.386)  CM vs control: OR 1.306 (95% CI 1.048–1.627)  BI vs control: OR 1.064 (95% CI 1.003–1.128)  Psychotherapy + BI vs control: OR 1.500 (95% CI 1.055–2.133) |
| Hettema, 2005 | MI vs control  Quantity: d_c_ 0.30 (95% CI 0.09, 0.52; p <0.05)  Frequency: d_c_ 0.31 (95% CI 0.18, 0.44; p <0.05)  Blood alcohol concent: d_c_ 0.22 (95% CI 0.10, 0.34; p <0.05) |
| Kelly, 2020 | Continuous abstinence: Manualised AA/TSF superior to other clinical interventions (e.g. CBT) at 12 months (RR 1.21; 95% CI 1.03,1.42), 24 months (RR 1.37, 95% CI 1.04,1.82) and 36 months (RR 1.42, 95% CI 1.17, 1.73).  Percentage days abstinent: Manualised AA/TSF superior to other clinical interventions at 24 months (MD 12.91; 95% CI 7.55, 18.29) and 36 months (MD 6.64; 95% CI 1.54, 11.75). |
| Kownacki, 1999 | No |
| Powers, 2008 | Medium effect size, Hedges' g = 0.55 |
| Ray, 2020 | No |
| Riper, 2014 | CBT/MI superior to control effective in AUD and major depressive disorder compared with controls, with small overall effect size (g = 0.17; 95% CI 0.07-0.28; p <0.001) for decrease in alcohol consumption. For face-to-face interventions the effect size is g = 0.16; 95% CI 0.05-0.28); p <0.01. |
| Roozen, 2004 | Number of drinking days: CRA superior to usual care (weighted mean difference −0.94; 95% CI −1.60 −0.27) |
| Roozen, 2006 | No |

AA, Alcoholics Anonymous; AUD, alcohol use disorders; BI, brief interventions; CBT, cognitive behaviour therapy; CI, confidence interval; CM, contingency management; CRA, Community Reinforcement Approach; MD, mean difference; MI, motivational interviewing; OR, odds ratio; RoB, risk of bias; RR, risk ratio; TSF, 12-step facilitation.
